# Within-person pain variability and physical activity in older adults with osteoarthritis from six European countries

**DOI:** 10.1186/s12891-018-2392-0

**Published:** 2019-01-05

**Authors:** Erik J. Timmermans, Elisa J. de Koning, Natasja M. van Schoor, Suzan van der Pas, Michael D. Denkinger, Elaine M. Dennison, Stefania Maggi, Nancy L. Pedersen, Ángel Otero, Richard Peter, Cyrus Cooper, Paola Siviero, Maria Victoria Castell, Florian Herbolsheimer, Mark Edwards, Federica Limongi, Dorly J. H. Deeg, Laura A. Schaap

**Affiliations:** 1 Department of Epidemiology and Biostatistics, Amsterdam University Medical Center, VU University Medical Center, Amsterdam Public Health Research Institute, De Boelelaan 1089A, 1081HV, Amsterdam, Netherlands; 2 Geriatric Research Unit / Institute of Epidemiology and Medical Biometry, AGAPLESION Bethesda Hospital, Ulm University, Ulm, Germany; 3 MRC Lifecourse Epidemiology Unit, University of Southampton, Southampton General Hospital, Southampton, UK; 4 National Research Council, Aging Branch, Institute of Neuroscience, Padova, Italy; 5 Department of Medical Epidemiology and Biostatistics, Karolinska Institute, Stockholm, Sweden; 6 Department of Preventive Medicine and Public Health, Unit of Primary Care and Family Medicine, Faculty of Medicine, Universidad Autonoma de Madrid, Madrid, Spain; 7 Institute of the History, Philosophy and Ethics of Medicine, Ulm University, Ulm, Germany; 8 Department of Health Sciences, Faculty of Science, Amsterdam Public Health Research Institute, Vrije Universiteit Amsterdam, Amsterdam, Netherlands

**Keywords:** European multi-cohort study, Older adults, Osteoarthritis, Pain variability, Pain severity, Physical activity

## Abstract

**Background:**

This study examines the association of both pain severity and within-person pain variability with physical activity (PA) in older adults with osteoarthritis (OA).

**Methods:**

Data from the European Project on OSteoArthritis were used. At baseline, clinical classification criteria of the American College of Rheumatology were used to diagnose OA in older adults (65–85 years). At baseline and 12–18 months follow-up, frequency and duration of participation in the activities walking, cycling, gardening, light and heavy household tasks, and sports activities were assessed with the Longitudinal Aging Study Amsterdam Physical Activity Questionnaire. Physical activity was calculated in kcal/day, based on frequency, duration, body weight and the metabolic equivalent of each activity performed. At baseline and 12–18 months follow-up, pain severity was assessed using the pain subscales of the Western Ontario and McMaster Universities OA Index and the Australian/Canadian Hand OA Index. Within-person pain variability was assessed using two-week pain calendars that were completed at baseline, 6 months follow-up and 12–18 months follow-up.

**Results:**

Of all 669 participants, 70.0% were women. Sex-stratified multiple linear regression analyses showed that greater pain severity at baseline was cross-sectionally associated with less PA in women (Ratio = 0.95, 95% CI = 0.90–0.99), but not in men (Ratio = 0.99, 95% CI = 0.85–1.15). The longitudinal analyses showed a statistically significant inverse association between pain severity at baseline and PA at follow-up in women (Ratio = 0.94, 95% CI = 0.89–0.99), but not in men (Ratio = 1.00, 95% CI = 0.87–1.11). Greater pain variability over 12–18 months was associated with more PA at follow-up in men (Ratio = 1.18, 95% CI = 1.01–1.38), but not in women (Ratio = 0.94, 95% CI = 0.86–1.03).

**Conclusions:**

Greater pain severity and less pain variability are associated with less PA in older adults with OA. These associations are different for men and women. The observed sex differences in the various associations should be studied in more detail and need replication in future research.

## Background

Osteoarthritis (OA) is one of the most common musculoskeletal disorders worldwide [1]. The condition is a leading cause of disability and a major contributor to loss of independence among older adults, with joint pain representing one of the key debilitating symptoms [1–3]. While OA is characterized as a slowly progressive disease, acute flares, episodes of severe pain, and substantial fluctuations in pain intensity are part of the natural course of the condition [4, 5]. It has been suggested that physical activity (PA) reduces joint pain in older adults with OA, and helps them to maintain or improve functioning [6–10]. Despite the potential health benefits of PA are well established, the majority of older people with OA are not sufficiently physically active [11–16]. Besides the fear of continuing or worsening wear and tear within the joint, it has been suggested that the most important reason for older adults with OA to avoid physical activities is pain that is associated with initiating activities [15, 17–19].

Pain patterns differ substantially across individuals with OA [4, 5, 20, 21]. Several studies have shown that higher levels of pain severity are associated with less PA in older people with OA [15, 18, 19, 22, 23]. Although joint pain often fluctuates in older adults with OA, previous studies mainly focused on the impact of momentary joint pain severity on PA and did not investigate the association between joint pain variability and PA in these individuals.

The fluctuating course of joint pain in OA requires repeated measurements and is difficult to capture in research based on commonly used extensive instruments, such as the Western Ontario and McMaster Universities OA Index (WOMAC) and the Australian/Canadian Hand Osteoarthritis Index (AUSCAN) [24–26]. Pain variability can more easily be measured using pain calendars (i.e., pain diaries) over a large period of time. In the European Project on OSteoArthritis (EPOSA), WOMAC, AUSCAN as well as pain calendars have been used to assess pain [27]. The EPOSA study provides a unique opportunity to assess the association of both pain severity and within-person pain variability with PA in older adults with OA in the general population across six European countries, including both older adults with mild and severe OA, and those seeking care and not seeking care [27]. Assuming that a lack of predictability of pain is detrimental to PA [18], it is hypothesized that greater pain severity and greater pain variability over time is associated with less PA in these individuals.

## Methods

### Design and study sample

In the present study, data from the European Project on OSteoArthritis (EPOSA) were used. The main objective of the EPOSA study is to investigate the personal and societal burden of OA and its determinants in older adults in six European countries. The study design and data collection are described in detail elsewhere [27]. In summary, random samples were taken from existing population-based cohorts in five European countries (Germany, the Netherlands, Spain, Sweden, and the United Kingdom). In Italy, a new sample was drawn. In the EPOSA study, the data collection took place twice with 12–18 months between the baseline and the follow-up measurement. At baseline and 12–18 months follow-up, a total of 2942 (average response rate: 72.8% (range: 64.6–82.2%)) and 2455 (average response rate: 83.4% (range: 68.2–92.3%)) older adults, respectively, participated in the EPOSA project. All participants were interviewed by a trained interviewer at home or in a clinical center, using a standardized questionnaire and a clinical exam. After the baseline and 12–18 months follow-up interview as well as 6 months after the baseline interview, participants were asked to complete a two-week calendar assessing joint pain. At baseline, the classification criteria of the American College of Rheumatology were used to diagnose clinical knee, hand and hip OA [28]. In total, 889 participants had knee, hand and/or hip OA at baseline. Of all participants with OA at baseline, 713 individuals also participated at follow-up. The final analytical sample consisted of 669 older adults with clinical OA who participated at baseline as well as at follow-up and did not report joint replacements at the site of the clinical OA. The study design and all procedures were approved by the Ethical Review Boards of the participating institutions. All participants provided written informed consent prior to the start of the study.

### Measurements

#### Dependent variable

##### Physical activity

At baseline and 12–18 months follow-up, physical activity (PA) was assessed using the Longitudinal Aging Study Amsterdam Physical Activity Questionnaire (LAPAQ) [29]. The LAPAQ examines the frequency and duration of participation in different activities (i.e., walking outside, cycling, gardening, light and heavy household tasks, and a maximum of two sports) during the previous two weeks. Physical activity was calculated in kcal/day, based on frequency, duration, body weight and the metabolic equivalent of each performed activity.

#### Independent variables

##### Pain severity

At baseline and 12–18 months follow-up, pain severity in the past 48 h was measured with the WOMAC (knee and hip OA) and the AUSCAN (hand OA) pain subscales. The WOMAC and AUSCAN pain subscales are described in detail elsewhere [24, 25]. Because these pain subscales are scored on the same scale (score range: 0–20), the scores of knee, hip and hand pain were added up, resulting in a total joint pain severity score (score range: 0–60). For comparability to other studies [24–26], this WOMAC/AUSCAN combined pain score was transformed to a 0–100 scale. A higher WOMAC/AUSCAN combined pain score indicates more joint pain.

##### Pain variability

Pain variability was assessed with two-week pain calendars completed by participants immediately after the baseline interview, six months later and after the 12–18 months follow-up interview. Participants from Spain did not complete the pain calendar at 6-months follow-up. Per day, participants indicated how much joint pain they experienced on an 11-point rating scale (score range: 0–10). On this rating scale, a score of 0 represents no pain and a score of 10 indicates extreme pain. For each participant, the standard deviation (SD) of the scores from all available days of the three calendars was used as measure of individual day-to-day pain variability. A larger individual SD score indicates more day-to-day pain variability within a person. At least 7 of 14 days of at least one calendar had to be completed by a participant in order to be included in the pain variability analyses. This approach corresponds with previous research [26].

##### Potential effect modifiers and confounders

Age, sex (0 = men, 1 = women), and country of residence were considered as potential effect modifiers in the association between pain and PA [30–32]. Age was categorized into younger-old people and older-old people on the basis of 1–2 quartiles (65.0–74.0 years) and 3–4 quartiles (74.1–85.0 years), respectively. Country of residence was dummy-coded and included: Germany, Italy, the Netherlands, Spain, Sweden, and the United Kingdom. Sweden was considered as the reference category, because the Swedish respondents reported, on average, to be physically less active.

The following potential confounders were considered in this study: age in years, sex, country of residence (if no effect modifiers), educational level, smoking, alcohol consumption, weight status, number of chronic diseases, and use of pain medications.

Educational level was classified into four categories: no education (reference category; elementary school not completed), low (elementary school completed), intermediate (vocational or general secondary education), and high (college or university education).

Smoking status was self-reported and classified into three categories: never smokers (reference category), former smokers, and current smokers. Alcohol consumption was self-reported (0 = no, 1 = yes).

Weight status was assessed using the Body Mass Index, which was calculated as weight in kilograms divided by height in meters squared [33]. The weight of participants was measured to the nearest 0.1 kg using a calibrated scale. The height of participants was measured to the nearest 0.001 m using a stadiometer.

Number of chronic diseases was assessed by asking for the presence of the following chronic diseases: chronic non-specific lung disease, cardiovascular diseases, peripheral artery diseases, diabetes mellitus, stroke, cancer, and osteoporosis.

Medication use (0 = no, 1 = yes) referred to the use of analgesics (Anatomical Therapeutic Chemical classification: N02 subgroup) and/or anti-inflammatory and anti-rheumatic products (Anatomical Therapeutic Chemical classification: M01 subgroup).

##### Statistical analyses

Characteristics of the study sample were presented using descriptive statistics. Means and SDs were presented for normally distributed continuous variables. Medians and interquartile ranges (IQRs) were presented for skewed continuous variables. Frequencies and proportions were presented for dichotomous or categorical variables. With the exception of age, sex and country, all descriptive statistics were weighted to adjust for differences in the distributions of age and sex across the samples of the six cohort studies. Weights were calculated per sex and per five-year age category, using the formula W=N_exp_/N_obs_, where N_exp_ is the number of persons in a specific age/sex category in the European population and N_obs_ is the number of persons in a specific age/sex category in the cohort [26, 34].

Multiple linear regression analyses were used to examine the associations between each pain measure and PA. To study possible effect modification by age, sex, and country of residence, an interaction term of the determinant with the potential effect modifier was created. This interaction term was added to the model. If the *p*-value of the interaction term was below 0.10, effect modification was considered to be present and stratified analyses were conducted [35]. In case it turned out that age, sex or country of residence was no effect modifier, the variable was considered as confounder in the subsequent analyses.

The associations between the pain measures and PA were constructed step by step in two models. In Model 1, we adjusted for age, sex, and country of residence (if no effect modifiers). In Model 2, we additionally adjusted for educational level, smoking, alcohol consumption, weight status, number of chronic diseases, and use of medications.

For pain severity, both cross-sectional and longitudinal multiple linear regression analyses were conducted. For the cross-sectional analysis, the baseline WOMAC/AUSCAN combined pain score was the main determinant and PA at baseline the continuous outcome measure. To examine the prospective association between pain severity and PA, the baseline WOMAC/AUSCAN combined pain score was the main determinant and PA at follow-up the outcome measure. In this analysis, PA at baseline was used as additional covariate.

To study whether pain fluctuations over time affect PA, multiple linear regression analyses were conducted with the individual joint pain SDs (calculated from the three two-week pain calendars at baseline, 6 months follow-up and 12–18 months follow-up) as the main determinant and PA at 12–18 months follow-up as outcome measure.

Three pre-planned sensitivity analyses were conducted. A potential disadvantage of using the SD of the individual pain intensity scores from all available pain calendar days as measure of day-to-day pain variability is that the SD is the same for individuals who report the same extent of fluctuations, regardless of their mean pain intensity score [26]. For example, two persons with mean pain intensity levels of 0 and 10, respectively, who experience no pain fluctuations, will both have a SD of 0. In the first pre-planned sensitivity analysis, we repeated the pain variability analysis whilst leaving out persons with no or only little reported joint pain (mean pain score on at least one calendar ≤2).

Hip and knee OA might be more strongly related to the physical activities that are assessed by the LAPAQ. In the second pre-planned sensitivity analysis, we repeated all analyses whilst leaving out persons with only hand OA. It is expected that these analyses reveal stronger associations of both pain severity and within-person pain variability with PA than those in which also participants with hand OA are included.

In the present study, the pain calendar at 12–18 months follow-up was completed in the two weeks after the PA assessment. However, we defined PA as our dependent variable. In the third pre-planned sensitivity analysis, we repeated the pain variability analyses whilst leaving out the 12–18 months pain calendar.

Because the distribution of PA was skewed to the right, we performed a natural log transformation on the LAPAQ data (i.e., Ln(1 + LAPAQ score) in the present study. The regression coefficients and confidence intervals (CIs) from the multiple linear regression analyses were back-transformed to obtain interpretable ratios. From these ratios, a percentage of change in the outcome variable (PA) per one-unit change in the determinant (pain) can be derived. Because a one-unit change on the pain severity scale of 0–100 is very small, we derived a more meaningful 10-unit change in the present study [36]. A two-sided *p*-value below 0.05 was considered as statistically significant. All analyses were conducted with IBM SPSS Statistics for Windows, Version 22.0 [37].

## Results

The characteristics of the study sample are presented in Table 1. The mean age of all 669 participants at baseline was 73.9 (SD = 4.9) years with an age-range of 65–85 years. The majority (70.0%) of participants was woman. Of all 669 participants, 62.9% had knee OA, 19.9% had hip OA, and 56.5% had hand OA. At baseline and 12–18 months follow-up, the average pain severity was 18.1 (SD = 12.3) and 17.9 (SD = 15.0) on the WOMAC/AUSCAN combined pain score, respectively. The participants completed, on average, 34.2 (SD = 9.2) (range = 7–42 days) days of the pain calendars. In the full sample, the average pain variability on the pain calendars was 1.4 (SD = 0.8) individual standard deviation units. At baseline and 12–18 months follow-up, the participants spent 691.5 (IQR = 437.4–1043.4) and 664.0 (IQR = 417.5–1077.3) kcal/day on PA, respectively (Table 1).

**Table 1:** Characteristics of the study sample^a,b^

	Study sample (*n* = 669)	Men (*n* = 211)	Women (*n* = 458)
Age (in years) (Mean (SD))	73.9 (4.9)	74.5 (5.1)	73.7 (4.8)
Country (%)			
Germany	8.4	9.5	7.9
Italy	17.6	19.9	16.6
Netherlands	16.6	14.7	17.4
Spain	21.8	20.4	22.5
Sweden	20.6	16.1	22.7
United Kingdom	14.9	19.4	12.9
Educational level (%)			
No education	14.4	12.0	15.3
Low	35.4	35.1	35.5
Intermediate	32.3	35.7	31.0
High	17.9	17.2	18.2
Smoking (%)			
Never	52.4	29.6	61.2
Former	41.9	63.9	33.5
Current	5.7	6.5	5.3
Alcohol consumption (yes) (%)	70.5	86.1	64.5
Weight status (BMI in kg/m^2^) (Mean (SD))	28.5 (5.0)	28.4 (3.9)	28.5 (5.4)
Number of chronic diseases (Median (IQR))	1 (0–2)	1 (0–2)	1 (0–2)
Use of analgesic and/or anti-inflammatory and anti-rheumatic products (yes) (%)	32.7	33.0	32.6
Presence of clinical osteoarthritis (%)			
Knee osteoarthritis	62.9	67.8	61.0
Hand osteoarthritis	56.5	42.7	61.9
Hip osteoarthritis	19.9	19.3	20.2
Pain severity at baseline (Mean (SD)) ^c^			
Baseline	18.1 (12.3)	14.6 (9.8)	19.4 (12.9)
Follow-up	17.9 (15.0)	13.7 (13.4)	19.6 (14.4)
Pain variability (Mean (SD)) ^d^	1.4 (0.8)	1.4 (0.9)	1.4 (0.8)
Physical activity (in kcal/day)			
(Median (IQR))			
Baseline	691.5 (437.4–1043.4)	636.6 (383.1–1090.1)	704.0 (447.7–1030.4)
Follow-up	664.0 (417.5–1077.3)	662.4 (348.0–1146.2)	667.1 (436.8–1059.1)

^a^Abbreviations: *BMI* Body Mass Index, *IQR* Interquartile range, *n* Number of participants, *kcal* Kilocalories, *kg/m*^*2*^ kilogram per square meter, *OA* Osteoarthritis, *SD* Standard deviation

^b^With exception of age and country, descriptive statistics are weighted

^c^A higher pain severity score indicates more pain

^d^Pain variability is presented in individual standard deviation units. A higher pain variability score indicates more day-to-day pain fluctuations

In all analyses, sex was found to be a significant effect modifier and, therefore, all analyses were stratified for sex. Age and country of residence were not found to be a significant effect modifier in all analyses.

### Cross-sectional analyses: pain severity and physical activity

After adjustment for age and country of residence, the cross-sectional baseline association between pain severity and PA was statistically significant in women (Ratio = 0.94, 95% CI = 0.89–0.98) (Table 2; Model 1). After additional adjustment for all other confounders, the association between pain severity and PA remained statistically significant in women (Ratio = 0.95, 95% CI = 0.90–0.99). Accordingly, a 10-unit higher pain severity score is associated with 5% less PA (Table 2; Model 2). No statistically significant association was observed between pain severity and PA in men (Table 2).

**Table 2:** Cross-sectional associations between pain severity and physical activity, stratified by sex^a-d^

	Men		Women	
	Ratio (95% CI)	*p*-value	Ratio (95% CI)	*p*-value
Model 1	1.00 (0.86–1.16)	0.97	0.94 (0.89–0.98)	0.02
Model 2	0.99 (0.85–1.15)	0.87	0.95 (0.90–0.99)	0.04

^a^Abbreviation: *CI* Confidence Interval

^b^In these analyses, the baseline WOMAC/AUSCAN combined pain score was the main determinant and physical activity at baseline the outcome measure

^c^Model 1: adjusted for age and country of residence

Model 2: additionally adjusted for educational level, smoking, alcohol consumption, weight status, number of chronic diseases, and use of analgesics and/or anti-inflammatory and anti-rheumatic products

^d^The cross-sectional associations are displayed per 10 units increase in pain severity

### Longitudinal analyses: pain severity and physical activity

After adjustment for age, country of residence and PA at baseline, the prospective association between pain severity at baseline and PA at 12–18 months follow-up was statistically significant in women (Ratio = 0.94, 95% CI = 0.89–0.99) (Table 3; Model 1). After additional adjustment for all other confounders, this association remained unchanged in women (Ratio = 0.94, 95% CI = 0.89–0.99) (Table 3; Model 1). Accordingly, a 10-unit higher pain severity score at baseline is associated with 6% less PA at follow-up in women. The prospective association between pain severity at baseline and PA at 12–18 months follow-up was not statistically significant in men (Table 3).

**Table 3:** Longitudinal associations between pain severity and physical activity, stratified by sex^a-d^

	Men		Women	
	Ratio (95% CI)	*p*-value	Ratio (95% CI)	*p*-value
Model 1	0.99 (0.88–1.11)	0.86	0.94 (0.89–0.99)	0.03
Model 2	1.00 (0.87–1.11)	0.81	0.94 (0.89–0.99)	0.02

^a^Abbreviation: *CI* Confidence Interval

^b^In these analyses, the baseline WOMAC/AUSCAN combined pain score was the main determinant and physical activity at 12–18 months follow-up the outcome measure

^c^Model 1: adjusted for age, country of residence, and physical activity at baseline

Model 2: additionally adjusted for educational level, smoking, alcohol consumption, weight status, number of chronic diseases, and use of analgesics and/or anti-inflammatory and anti-rheumatic products

^d^The longitudinal associations are displayed per 10 units increase in pain severity

### Longitudinal analyses: pain variability and physical activity

A greater pain variability over 12–18 months was significantly associated with more PA in men (Ratio = 1.18, 95% CI = 1.01–1.38), but with less PA in women although not significant (Ratio = 0.94, 95% CI = 0.86–1.03) (Table 4; Model 2). Accordingly, a 1-unit higher pain variability score is associated with 18% more PA in men, and with 6% less PA in women.

**Table 4:** Longitudinal associations between pain variability and physical activity, stratified by sex^a-c^

	Men		Women	
	Ratio (95% CI)	*p*-value	Ratio (95% CI)	*p*-value
Model 1	1.15 (0.98–1.33)	0.08	0.93 (0.85–1.02)	0.14
Model 2	1.18 (1.01–1.38)	0.04	0.94 (0.86–1.03)	0.21

^a^Abbreviation: *CI* Confidence Interval

^b^In these analyses, the individual joint pain standard deviation score (calculated from the three two-week pain calendars at baseline, 6 months follow-up and 12–18 months follow-up) was the main determinant and physical activity at 12–18 months follow-up the outcome measure

^c^Model 1: adjusted for age and country of residence

Model 2: additionally adjusted for educational level, smoking, alcohol consumption, weight status, number of chronic diseases, and use of analgesics and/or anti-inflammatory and anti-rheumatic products

### Sensitivity analysis

The various sensitivity analyses did not change any of the conclusions (results not shown).

## Discussion

This study examined whether pain severity and pain fluctuations over time were associated with PA in older adults with OA from the general European population. The results showed that greater pain severity was associated with less PA in women, but not in men. Furthermore, the findings showed that greater pain variability was associated with more PA in men. Pain variability was not associated with PA in women. The various sensitivity analyses did not result in substantially different findings.

The findings of this study showed that greater pain severity was associated with less PA in older women. The observed difference in this association between men and women could be possibly explained by differences in coping strategies that are used by men and women [38]. Pain catastrophizing is a pain coping strategy referring to the exacerbation of pain-related information [38, 39], while self-efficacy refers to the belief of a person in his or her ability to achieve goals [38, 40]. Previous studies have shown that catastrophizing is associated with pain and pain-related disability and that women use this coping strategy more often than men [38, 41]. Previous studies have also shown that men more often demonstrate higher levels of self-efficacy than women [38, 42]. Furthermore, the observed sex-differences in the association between pain severity and PA could be explained by socio-cultural beliefs or gender role expectations regarding how men and women should respond to pain. It might be less accepted from men than from women when they adjust their PA levels because of greater pain severity [38, 43]. The influencing role of different coping strategies in the association between pain and PA in older men and women with OA should be addressed in future research.

The results of the present study showed that greater pain variability was associated with more PA in older men. On forehand, we expected that greater pain variability would mean more unpredictability, which may lead to fear of sudden increases in pain, that would lead individuals to reduce their activities and activity levels [18]. A possible explanation for the current finding could be that older men with OA, in periods of less pain, become considerably more physically active, until pain increases again. As a result, more pain fluctuations could lead to more PA.

Although the results of the present study suggest that pain severity and pain variability affect PA levels of older adults with OA, it could also be that PA affects pain [7, 9]. It has been suggested that physical activity (e.g., supervised exercise therapy) could strengthen the muscles around an affected joint, which helps to stabilize the joint and to absorb most of the forces presented to the joint, resulting in less pain [44]. Future research is needed to consider the possibility of reversed causality, by examining how PA at baseline influences pain at follow-up and what the potential implications of this relationship are for PA at follow-up.

The EPOSA-project is a large-scale study with a pre-harmonized dataset including prospective cohort data from older adults with clinical knee, hand and/or hip OA from six European countries [27]. An important strength of our study is that the assessment of clinical OA was performed according to a uniform protocol in all six countries. Another strength is that the study sample was drawn from the general population. Consequently, our study includes data from individuals with mild and severe OA. In addition, our study includes data from persons seeking care and not seeking care. This increases generalizability of our results. Furthermore, a strength of this study is the assessment of day-to-day pain variability using several two-week pain calendars over a large time period. This is a novel and promising way of investigating joint pain in OA [26].

The current study has some limitations as well. Firstly, the participants in our study were not asked about other types of pain that they may have experienced at the same time as their joint pain. As a consequence, we were unable to control for this potentially important confounder. Secondly, there was no exact overlap between the timing of the pain assessments and the timing of the PA assessments. In the present study, the pain calendar at 12–18 months follow-up was completed in the two weeks after the PA assessment, whereas PA was our dependent variable. However, the pain calendar started the day after the 12–18 months follow-up interview, and consequently, the time period between these assessments was quite short. Excluding data from the third pain calendar would not only reduce the statistical power of our analyses, but would also mean that PA would be assessed six months after the last pain calendar (and 12–18 months for Spain), which is a much longer period. The sensitivity analyses in which the 12–18 months pain calendar was left out showed similar results as the main analyses. Thirdly, the SD of the individual pain scores from all available days of three two-week pain calendars was used as measure of within-person pain variability in this study. It has been suggested that these SD scores may suffer from relatively low reliability [45], and this might be more pronounced if the number of observations is low. In the current study, we combined three two-week pain calendars in order to make the reliability of the pain variability measure as high as possible. For the majority of the participants, the individual SD was calculated from 42 observations. Finally, PA was self-reported using the LAPAQ [29]. It has been suggested that self-reported PA has some limitations compared to objectively measured PA, such as susceptibility to overestimation, recall, and culture difference bias, especially in older adults [46, 47].

Overall, the current study may help to better understand pain patterns in older adults with OA and how these patterns affect daily functioning of these individuals in terms of PA. However, the observed sex differences in the various associations should be studied in more detail and need replication in future research. Ultimately, health care professionals could use the insights gained from such studies in clinical encounters to tailor recommendations for the timing of pain medication use and PA behavioral strategies.

To obtain more detailed knowledge about the association between OA-related pain and PA, future research could objectively assess PA using accelerometers and let participants fill in a pain calendar at the same time. Furthermore, these studies could focus on whether pain variability is associated with specific activities in men and women, and whether there are differences in other factors that may cause sex differences in the various associations.

## Conclusions

In conclusion, the present study showed that greater pain severity was associated with less PA in older women. Furthermore, the findings of this study show that greater pain variability was associated with more PA in older men.

## Abbreviations

AUSCAN: Australian/Canadian Hand Osteoarthritis Index (AUSCAN)
CI: Confidence interval
EPOSA: European Project on OSteoArthritis
IBM SPSS: International Business Machines Corporation Statistical Package for the Social Sciences
IQR: Interquartile range
kcal: Kilocalories
kg/m^2^: Kilogram per square meter
LAPAQ: Longitudinal Aging Study Amsterdam Physical Activity Questionnaire
Ln: Natural logarithm
n: Number of participants
N_exp_: Number of persons in a specific age/sex category in the European population
N_obs_: Number of persons in a specific age/sex category in the cohort
OA: Osteoarthritis
PA: Physical activity
SD: Standard deviation
W: Weight
WOMAC: Western Ontario and McMaster Universities OA Index
